# Renin Angiotensin system-modifying therapies are associated with improved pulmonary health

**DOI:** 10.1186/s40842-017-0044-1

**Published:** 2017-06-28

**Authors:** Maira Soto, Soo I. Bang, Jeff McCombs, Kathleen E. Rodgers

**Affiliations:** 1 Titus Family Department of Clinical Pharmacy, School of Pharmacy, University of Southern California, 1985 Zonal Ave.,PSC 530, Los Angeles, CA 90033 USA; 2 Department of Pharmaceutical and Health Economics, School of Pharmacy, University of Southern California, 635 Downey Way, VPD 212B, Los Angeles, CA 90089 USA

**Keywords:** Diabetic complications, Claims data, Pulmonary outcomes, Lung disease, Ace-I, Arb

## Abstract

**Background:**

Pulmonary diseases are often complicated and have diverse etiologies. One common factor is the lack of therapeutics available for these diseases. The goal of this study was to investigate the impact of Renin-Angiotensin System (RAS)-modifying medications on incidence and time to pulmonary complications.

**Methods:**

A retrospective analysis was conducted using claims data from a US commercial insurance company (2007–2013). The study consisted of patients with an emerging hypertension (HTN) diagnosis. Cox analysis was used to look at the effect of angiotensin converting enzyme inhibitors (ACE-Is) and angiotensin receptor blockers (ARBs) in this population. The events included pneumonia and influenza (infectious), Chronic obstructive pulmonary disease (COPD) and allied conditions (inflammatory), and other diseases (structural).

**Results:**

A total of 215,225 patients were followed in the study. These fell into three groups depending on the first prescribed anti-hypertension medication; ACE-Is (47.21%), ARBs (11.40%) and calcium channel blockers (CCBs)/Diuretics-Control (41.39%). The use of ACE-I as first treatment significantly reduced the incidence of infectious (Hazard Ratio (HR) 0.886, 95% Confidence Interval (95% CI) 0.859–0.886), inflammatory (HR 0.924, 95% CI 0.906–0.942) and structural outcomes (HR 0.865, 95% CI 0.847–0.885); it also increased the time (delayed) to diagnosis with prolonged treatment. Primary ARB use only significantly lowered the incidence of structural outcomes (HR 0.900, 95% CI 0.868–0.933); prolonged treatment did reduce incidence of all three diagnosis groups and significantly delayed disease onset.

**Conclusions:**

There is an association between the use of ACE-Is and ARBs and a delay in the progression of pulmonary complications in vulnerable populations. Research into the RAS may identify future therapies for patients with potential chronic pulmonary conditions.

## Background

Lung tissue has some of the most delicate architecture in the body. Small structural changes can have a large impact on its functional ability [1]. Fibrosis decreases the lungs’ ability to properly conduct gas exchange and can increase chance of infection [2–5]. Unfortunately, there are countless triggers that can be responsible for pulmonary disease, each etiology resulting in slightly different pathology and different treatment options [6–8].

Traditional studies have looked at the role of the Renin Angiotensin System (RAS) pathway in blood pressure regulation. We now know that modifiers of RAS may directly impact patient health beyond hypertension (HTN) regulation. Activation of the pathological arm of the RAS by angiotensin II (AngII) results in increased insulin resistance, oxidative stress (OS), chronic inflammation, HTN, and end organ failure [9–12]. Chronic inflammation and OS may contribute to the development of lung fibrosis through increasing tissue injury and reduces the ability of the lung to respond to additional insults such as infection.

Many of the drugs used to control HTN act to either reduce the production of AngII (angiotensin converting enzyme inhibitors [ACE-Is]) or block its action on it pathological receptor, AT1 (angiotensin receptor blockers [ARBs]). Through the blockade of AngII signaling, these drugs reduce OS and inflammation [13, 14]. Both ACE-I and ARBs have shown to be effective treatments in HTN, heart failure, diabetes and coronary heart disease [15]. Treatment with ACE-Is was shown to decrease the rate of cognitive and functional decline in patients with mild to moderate Alzheimer’s disease [16]. These actions, beyond the control of HTN, may contribute to the reduction in pulmonary diseases.

Our analysis employs data on pre-existing co-morbidities to better isolate the potential therapeutic benefit of targeting RAS to reduce lung complications using a retrospective cohort study of claims data. We evaluated the impact that RAS-modifying medications had on the pulmonary health of this population; including class of medication, calcium channel blocker (CCBs)/diuretic, ACE-I or ARB, and duration of drug exposure. The events included in the analysis are grouped as; pneumonia and influenza (infectious), COPD and allied conditions (inflammatory), and other diseases of the lung (structural). We limited our sample to patients with a diagnosis of HTN who initiated antihypertensive treatment during the data period to be able to calculate the impact of drug duration. Two models were then used to calculate the association between the type and duration of treatment of the initial HTN therapy prescribed on pulmonary outcomes. Even small improvements in the pulmonary health due to RAS-modifying drugs can validate this system as a potential target for developing novel therapies for vulnerable populations.

## Methods

### Study population

The Humana dataset, a United States-based insurance claims dataset, holds de-identified patient records for over 20 million patients for a 6.5 year period for which the data were available (1/1/2007–6/30/2013). Patient claims data were extracted for all patients with a recorded HTN diagnosis and who were initiated treatment with a first-line antihypertensive medications [ACE-Is, ARBs, CCBs or diuretics] during the data period (1/1/2007–6/30/2013). The date of the first HTN prescription claim was termed the index date and a patient episode was created based on the eligibility data, partitioning the patient’s paid claims into pre- and post-index periods.

### Inclusion/exclusion criteria

Patient were included in the study if the patients were continuously eligible for medical and pharmacy benefits for minimum of 6 months before and 12 months after the index date. Patient were excluded from the study when they met any one of the following criteria: (1) patients who were diagnosed with T2DM during the 12-months pre-index period, because patients with advanced diabetes are disproportionately prone to lung problems; (2) patients who were diagnosed with permanent lung conditions (ICD-9-CM codes 492–496 and 515–517) during the pre-index period, our focus is on emerging conditions; (3) patients who were diagnosed with HIV, cystic fibrosis, lung cancer, T1DM or received lung transplant at least once during the entire study period, these confounders may be difficult to control for in our model since it they are not well understood in terms of pulmonary outcomes.

### Study variables

The outcome variable was defined as the occurrence of the first pulmonary or lung complication diagnosis based on ICD-9-CM codes in the patient’s medical claims data. Pulmonary events included pneumonia and influenza (ICD-9-CM code 48×–infectious), COPD and allied conditions (ICD-9-CM code 49×–inflammatory), and other diseases (ICD-9-CM code 51×–structural). Time-to-event outcome variables were created based on the number of days between the index date and the first diagnosis event.

### Study design and strategy

This study is a retrospective cohort study of claims data from the HUMANA (Louisville, KY) health insurance company. To test the effect of RAS-modifying drugs on pulmonary outcomes the patient’s use of HTN medications was modeled in two ways (Figure 2a): (1) use of ACE-I, ARB or CCBs/diuretic, and (2) duration of RAS-modifying medication. Only patients who were prescribed a monotherapy were followed. The Initial drug model (Model 1) was tested to measure the extent to which the risk of the respiratory disease outcomes is impacted if the patient started are ACE-I or ARB medications relative to CCBs or diuretics. Duration model (Model 2) tested whether everyday use of HTN medications delays pulmonary outcomes. Episode length for each patient was calculated by summing up number of days with initial HTN medications until an outcome occurred. For patients without occurrence of an outcome, the length of the episode was defined as the number of days on initial HTN medications until the post-index period ends. A grace period of 30 days was used to define continuous usage of the treatment in order to allow for time between refills. Time to pulmonary health outcome is defined as the time from the start of treatment to the time ofearliest pulmonary disease outcome,. A subject is considered as censored if they did not experience an event by the end of follow-up.

### Covariates

Other covariates included age at the index date, gender, race, geographic regions, statin use, flu-vaccine use, T2DM and comorbidities. Comorbidity variables were created to capture the patient’s baseline mix of disease status which was categorized into 23 groups based on ICD-9-CM codes measured during the 6–12-months pre-index period.

### Statistical methods

Analyses were performed using SAS software, Version 9.3 of the SAS System for Unix. Cox proportional hazard analyses were performed for each drug effect model. Hazard ratios [HR] and 95% confidence interval [CI] were estimated with Cox proportional hazard model to accommodate differences on length of the post period data available for each patient using time-dependent covariates. Number-Needed-to Treat (NNT) was calculated by dividing the total number of incidents (patients that have been diagnosed with a pulmonary outcome after the start of treatment) for each treatment group by the cumulative years of drug exposure. Patients without diagnosis of each event during episode were considered censored at the end of the episode. Overall censoring is used when the data runs out before an event is observed. The termination of data for each patient is then used to calculate the length of the episode. Kaplan Meier curves for the survival function for incidence of infection, inflammatory and structural outcomes in the lung were generated using STATA (College Station, TX).

### Population

**Fig. 1:**
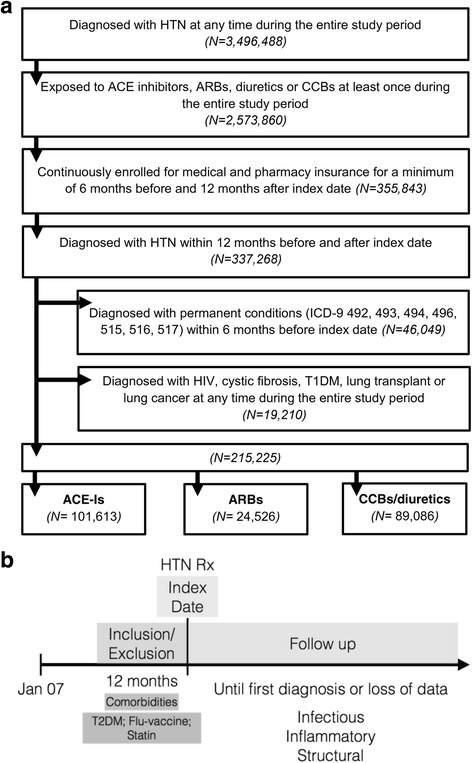
*Patient cascade chart and study design.* Selection criteria for HTN patients included in COX analysis study of the association between diabetes and pulmonary outcomes (**a**). Cox analysis was used to measure the association of first prescribed anti-hypertensive medication (ACE-Is and ARBs) with pulmonary diseases (**b**). The index date refers to the date the patient first filled a prescription for an anti-hypertensive medication. From there, and looking back 12 months, information on inclusion/exclusion criteria and incidence of comorbidities/co-variables was processed

Patients were selected initially if they were diagnosed with HTN during the data period (*N* = 3,496,488). This population was then required to have used one of four classes of anti-hypertensive medications used as initial therapy at least once during the entire study period. These selection criteria resulted in a cohort of 2,573,860 (Figure 1a). Episodes of care were defined based on the patients initial HTN prescription and episodes of treatment were included in the analysis if the patient was continuously enrolled for medical and pharmacy insurance for a minimum of 6 months before and 12 months after index date (*N* = 355,843) (Figure 1b). The requirement for HTN diagnosis during the pre-index date in the 12 months prior to the index date was met by 337,268 subjects. The final selection criteria eliminating patients with a prior chronic lung condition, as detected by ICD-9 codes 492, 493, 494, 496, 515, 516 and 517 (to allow evaluation of emerging chronic pulmonary diseases) as well as chronic conditions that are confounding factors hard to control for with regards to pulmonary outcomes (HIV, cystic fibrosis, lung transplant, lung cancer), type 1 diabetes at any time during the data period or T2DM within 12 months prior to the episode index date resulted in a final cohort of 215,225 patients. This group was further subdivided into three categories depending on the first prescribed HTN medication. These groups were patients taking; ACE-Is (*N* = 101,613), ARBs (*N* = 24,526) and the control group prescribed CCBs or diuretics (*N* = 89,086).

## Results

The patient demographics and comorbidity profiles of all patients, by HTN-medication subgroup, are displayed in Table 1. The study population is 52% female and the age distribution indicates that more than half the group in the age > 65. The rough similarity in the age distribution is likely to be a result of requiring a diagnosis of HTN as part of the inclusion criteria. Information on race was only available for Medicare Advantage Plan Part D members. Only 65% of the study population has data on race and over 85% of patients with recorded race data are white. Regional data between groups were included as a variable to control for regional practice patterns that may differ by geographical environment and culture. A majority of the data comes from patients residing in the South (63%) and Midwest (26.33%) regions of the country. Although baseline comorbidities were included in all analyses for the entire spectrum of diagnosis covered by ICD-9 codes, only those which most pertain to pulmonary diagnosis are included in Table 1. Patients receiving CCBs/diuretics (the control category) have the highest incidence of comorbidities at baseline highlighting the importance correcting for all baseline comorbidities in our Cox analyses.

**Table 1:** *Baseline characteristics of the population*

Variable	ACE-Is	ARBs	Control
Number of Subjects (%)	101,613 (47.2)	24,526 (11.4)	89,086 (41.4)
Sex (%)			
Female	47,195 (46.5)	12,550 (51.2)	52,189 (58.6)
Mean age (years ± SD)	62.91 (13.5)	62.05 (±13.3)	64.20 (±13.8)
Race (%)			
White	56,522 (55.6)	11,502 (46.9)	50,660 (56.9)
Black	5032 (5.0)	1531 (6.2)	7359 (8.3)
Others	2854 (2.8)	834 (3.4)	2432 (2.7)
Unknown	37,195 (36.6)	10,659 (43.5)	28,635 (32.1)
Comorbidities (ICD-9 codes)			
Infectious (001–139)	14,183 (14.0)	3299 (13.5)	15,016 (16.9)
Blood (280–289)	15,762 (15.5)	3858 (15.7)	18,105 (20.3)
AMI (410)	3081 (3.0)	308 (1.3)	1503 (1.7)
Angina pectoris (413)	4022 (4.0)	775 (3.2)	3594 (4.0)
Cardiac dysrhythmias (427)	13,591 (13.4)	2805 (11.4)	15,769 (17.7)
Heart failure (428)	4702 (4.6)	869 (3.5)	6859 (7.7)
Respiratory (460–519)	33,081 (32.6)	7995 (32.6)	28,836 (32.4)
Other Treatments			
Statin (%)	43,298 (42.6)	9393 (38.3)	33,043 (37.1)
Flu vaccine (%)	35,550 (35.0)	7914 (32.3)	32,498 (36.5)
T2D (%)	5423 (5.3)	1218 (5.0)	5281 (5.9)
Other Information			
Mean follow up time (days)	1663.08	1603.33	1664.05
STD (+/−)	593.43	611.68	599.37

Medicare (a small % of the Humana dataset) does not report patient race and will be reported as unknown here. Incidence of comorbidities in the 12 month pre-index period for all study groups. *Respiratory does not include codes covered by infectious, inflammatory or structural outcomes

**Fig. 2:**
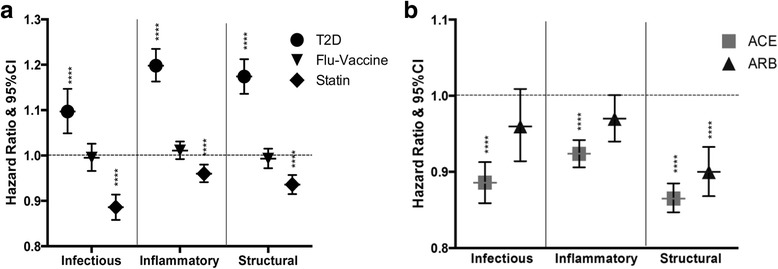
*Effect of RAS modifying drugs on pulmonary outcomes.* The association of diabetes diagnosis and incidence of infectious, inflammatory or structural outcomes events when compared to non-diabetic patients was measured using Cox analysis in this same model (**a**). The same parameters were measured using patients vaccinated for influenza compared to those who were not vaccinated and for patients taking statins at baseline compared to those not taking statins (**a**). These same analyses were done comparing the effect of initial choice of HTN therapy on infectious, inflammatory and structural outcomes; here we also corrected all of the baseline comorbidities; including T2DM, Flu-vaccine and statin use specifically (**b**). ****p < 0.0001

Diabetic patients are known to have a variety of complications, including those related to the lung. A Cox analysis was used to determine the association of T2DM with pulmonary outcomes in this population so that it could be corrected for in our further analysis (Figure 2a). The incidence of diagnosis of pulmonary outcomes was found to be greater in patients with a newly emerging T2DM diagnosis (infectious HR 1.097, 95% CI 1.049–1.147; inflammatory HR 1.198, 95% CI 1.163–1.235; structural HR 1.174, 95% CI 1.136–1.212).

The effect of influenza vaccination at baseline was measured in this population by measuring diagnosis incidence for patients who had a flu-vaccine as a baseline measure compared with those that were not vaccinated for influenza at baseline. Only the flu-vaccine was used since, unlike the vaccines against pneumonia which are administered every 5 years, it is a yearly vaccine for which we can capture adherence of the entire population at baseline (Figure 2a). Flu-vaccination had no significant effect on incidence of diagnosis (infectious HR 0.995, 95% CI 0.966–1.026; inflammatory HR 1.011, 95% CI 0.992–1.031; structural HR 0.993, 95% CI 0.972–1.015). As statins are shown to have anti-inflammatory effects [17], the effect of statin use at baseline was measured in the same model (Figure 2a) and was found to both decrease incidence (infectious HR 0.886, 95% CI 0.858–0.914; inflammatory HR 0.960, 95% CI 0.941–0.980; structural HR 0.936, 95% CI 0.915–0.957).

Model 1 was also used to look at the overall effects of ACE-Is or ARBs were compared with the effects of CCBs/diuretics (Figure 2b). Since we found a significant impact of T2DM, statin use and vaccine use, we included these parameters in this analysis to correct for their contribution. In this model, the Cox analysis showed that hypertensive patients taking ACE-Is were all significantly less likely to have a diagnosis of infectious, inflammatory and structural outcomes (HR 0.886, 95% CI 0.859–0.913; HR 0.924, 95% CI 0.906–0.942; HR 0.865, 95% CI 0.847–0.885). Patients taking ARBs were only less likely to be diagnosed with structural diseases (infectious HR 0.960, 95% CI 0.914–1.009; inflammatory HR 0.970, 95% CI 0.940–1.001; structural HR 0.900, 95% CI 0.915–0.957) in this model. We have also calculated the NNT in order to better understand the clinical significance of this data. On average 167 hypertensive patients must be placed on an ACE-I in order to reduce a pulmonary diagnosis for 1 patient. The clinical potential for the use of this drug is further exemplified by consideration of the number of hypertensive patients in the United States. According to Centers for Disease Control and Prevention, approximately 75 million people in the US have HTN [18]. Within this population 312,550–721,153 patients that may benefit from starting on an ACE-I for their HTN with regards to reduction in pulmonary outcomes. Further, this is a beneficial drug for the control of HTN and we are only looking at additional effects of a drug in a system for which it is not designed to treat pulmonary diseases. For ARBs, an average of 391 patients need to be treated in order to see an effect on pulmonary outcomes; this has a potential to reduce pulmonary diagnosis in 117,739–535,714 hypertensive patients.

**Fig. 3:**
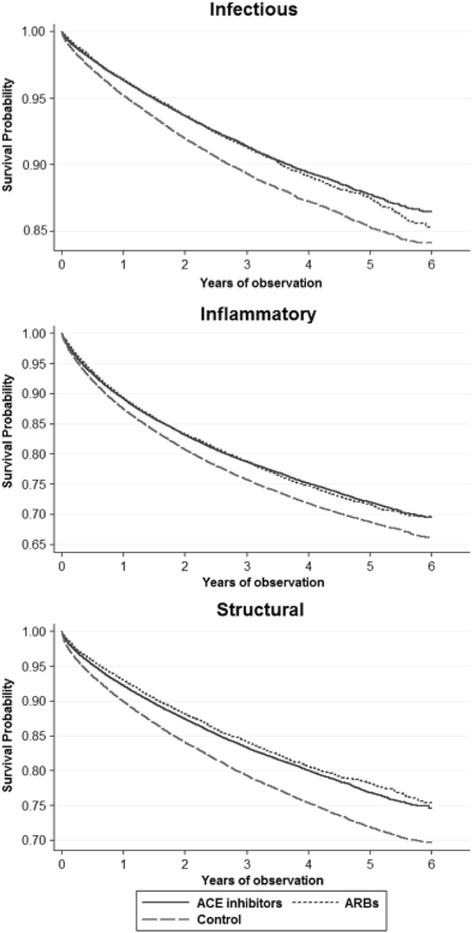
*Kaplan Meier curves for the survival function for incidence of infection, inflammatory and structural outcomes in the lung.* Figures were generated using STATA

Model 2 takes into consideration the duration of treatment used during Model 1. As in Model 1, we corrected for baseline comorbidities including T2DM, statin and vaccine use. Here, the Cox model, now expressed as likelihood of diagnosis per day of therapy (Table 2), calculated a beneficial effect on infectious, inflammatory and structural outcomes while taking any antihypertensive medication with continued drug use (Est −0.0020, −0.0016, −0.0015); this estimates the lower likelihood of disease diagnosis per day of treatment. Enhanced protection was noted if the anti-hypertensive medication was either an ACE-I (Est −0.0021, −0.0018, −0.0016) or ARB (Est −0.0030, −0.0017, −0.0021). These data are also displayed in a Kaplan Meier plot (Figure 3). This may signal that there is repair happening, not just prevention of further damage. The population in this study is also unlikely to discontinue the use of their medication, since they are taking ACE-Is or ARBs for HTN, and will likely have continued benefits as related to pulmonary outcomes.

**Table 2:** *Cox models were used to estimate the effect of different anti-hypertensive therapies on infectious, inflammatory and structural outcomes*

	Infectious (*N* = 21,523)	Inflammatory (*N* = 50,449)	Structural (*N* = 42,615)
ACE-I vs Control			
*Model 1 (Initial Drug)*			
HR	0.886	0.924	0.865
95%CI	0.859–0.886	0.906–0.942	0.847–0.885
*p*	<0.0001	<0.0001	<0.0001
NNT	240	156	104
*Model 2 (Drug duration)*			
Est	−0.0041	−0.0034	−0.031
*p*	<0.0001	<0.0001	<0.0001
ARB vs Control			
*Model 1 (Initial Drug)*			
HR	0.957	0.970	0.900
95%CI	0.914–1.009	0.940–1.001	0.868–0.933
*p*	0.1067	0.0568	<0.0001
NNT	637	395	140
*Model 2 (Drug duration)*			
Est	−0.0050	−0.0033	−0.036
*p*	<0.0001	<0.0001	<0.0001

Model 1 uses the first drug prescribed and follows the incidence of diagnosis up until the patient stops taking the medication. Model 2 takes into account the duration of the treatment in order to compare to emergence of disease. Diabetic-status, Flu Vaccine and Statin use are corrected for in each analysis comparing anti-hypertensive therapy. The number needed to treat (NNT) was also calculated for ACE-I and ARB use for infectious, inflammatory or structural outcomes

## Discussion

Pulmonary outcomes are an important part of a patient’s health and can have a huge impact on quality of life. Here we show that the use of RAS-modifying drugs for HTN is associated with improved lung health, this benefit is significantly improved by long-term use of these drug. Our study also documents that newly emerging T2DM is also an independent risk factor for lung diseases and these conditions have independent associations on patient morbidity and mortality; adding to the overwhelming evidence that the control and prevention of T2DM is a major public health issue and that clinicians need to add pulmonary issues to their long list of concerns when treating their diabetic patients. Statins were also shown to play a significant role in the overall pulmonary health of these patients.

HTN occurs in a substantial number of diabetic patients, so much so that it has been suggested that they share common disease mechanisms [19]. Pulmonary HTN (PHTN) is a disease closely associated with the both of these disease states and can contribute to pulmonary outcomes [20]. Although PHTN was not a specific focus in our studies we have corrected for these events under the comorbidity ‘Respiratory’.

In many studies ARBs and ACE-I have been shown to decrease OS and inflammation in hypertensive patients [21, 22]. Both these parameters can affect lung pathologies. The results in all analysis done here clearly show a benefit from RAS-modifying medications over other medications in reducing and delaying the incidences of infectious, inflammatory and structural outcomes. Although we cannot directly measure OS or inflammation in our patient population we can hypothesize that the protective effects we see here are likely due to similar changes in the lung caused by reducing the activation of AT_1_R.

There are important design and statistical limitations for this study that must be addressed. Data derived from claims data has the advantage of having very high numbers of patients, but, at the same time, the data are limited to diagnostic codes entered by physicians at the time the patient visits the hospital/clinic. Since we rely heavily on diagnosis made by doctors, we feel that grouping them in broader categories illuminates some of the coding differences that are attributed to physician preference. Lab values and questionnaire data are not available to us and so we cannot further stratify by some important parameters like BMI, smoker status or HbA1c levels. NNT is a value approximated from pooled HR data and is limited by factors such as the number of diagnoses in each group; it is provided only as an estimate of the effect on the population. We understand that these factors are very important, and have corrected for as much as possible given limitations of our dataset. It is important to note that the population size may be large enough to minimize some of these challenges, especially when the group most affected by HTN and pre-existing conditions (those taking ACE-I and ARBs as first line therapy as opposed to diuretics or CCBs as recommended [23]) is the group with the least amount of pulmonary complications. However, since these are not data derived from clinical trials, there is a chance that some important data may be missing from the patient files and should be used only as evidence of a possible new therapeutic target and not as a definite treatment.

Statistical methods are also critical to the validity of results derived from observational data. We also used a Cox analysis to account for any observed differences at the time of HTN treatment initiation that may be correlated with future pulmonary diseases. We observed significant increases in all pulmonary complications that were correlated with an emerging diabetic diagnosis controlling for a long list of baseline variables covering patient characteristics and co-morbidity status. These results are consistent hypotheses developed by authors Soto and Rodgers based on their laboratory studies documenting significant structural changes in the alveolar compartments and immune parameters of diabetic mice as well as the benefit from RAS-modifying therapies [24].

Finally, all studies in which patients are not randomized to the groups being compared are vulnerable to the criticism that an underlying *unobserved* factor correlated with T2DM, HTN and the outcomes of interest is confounding our results. While our analysis took significant care to include a long list of observable factors into the analysis, this criticism cannot be fully refuted. However, from a clinical perspective, our results highlight the positive effects of prescribing a RAS-modifying medication over another form of antihypertensive.

## Conclusion

These results are an important observation to those seeking to develop novel therapeutic approaches for vulnerable populations, like T2DM. RAS-modification may be key in regulating oxidative damage and chronic inflammation in patients with chronic illness.

## Abbreviations

ACE-I: ACE inhibitor
ARB: Angiotensin receptor blocker
BMI: Body mass index
CCB: Calcium channel blocker
COPD: Chronic obstructive pulmonary disease
HTN: Hypertension
MRSA: Methicillin resistant *S. aureus*
OS: Oxidative stress
RAS: Renin Angiotensin System
T1DM: Type 1 Diabetes Mellitus
T2DM: Type 2 Diabetes Mellitus
